# Light therapy for the treatment of delayed sleep-wake phase disorder in adults: a systematic review

**DOI:** 10.5935/1984-0063.20200074

**Published:** 2021

**Authors:** Jefferson Novais Gomes, Cristiane Dias, Renata Silva Brito, Juliana Rodrigues Lopes, Igor Alonso Oliveira, Alexandra Noemi Silva, Cristina Salles

**Affiliations:** Escola Bahiana de Medicina e Saúde Pública, Pesquisa - Salvador - Bahia - Brazil.

**Keywords:** Phototherapy, Sleep initiation and maintenance disorders, Circadian Rhythm, Chronobiology, Sleep-wake disorders, sleep

## Abstract

Delayed sleep-wake phase disorder (DSWPD) is characterized by sleep onset times, beyond the usual schedules and social conveniences, which potentially impacts on health as well as on school and professional performance. The most common treatment for DSWPD is the light administration (light therapy), through light devices, with or without behavioral instructions. Since there is no consensus in the literature about its efficacy and how it should be processed, this study aims to evaluate the light therapy effectiveness in the delayed sleep-wake phase disorder therapeutics. A systematic review was conducted using the MEDLINE/PubMed, Virtual Health Library Brazil, PsycINFO, Web of Science and Scopus databases along with a hand search until September 2020. The included studies presented participants diagnosed with insomnia or DSWPD, over 18-years old, treated only with morning light therapy, mentioning the light intensity (lux) used, and investigations with a control group. Studies reporting individuals with neurological or psychiatric disorders, shift-workers, or evaluating other sleep disorders were excluded. Among the 411 studies identified, five were selected for this review, resulting in a total sample of 140 individuals. Only two studies produced long-term results, showing that the benefits did not persist. In most studies, there were no statistically significant differences in the variables when comparing the intervention group and the control group. However, there were substantial clinical and laboratory advances in the sleep phase using light therapy when comparing phase advances for the same group concerning baseline values of sleep variables.

## INTRODUCTION

Delayed sleep-wake phase disorder (DSWPD) is characterized by a chronobiological disorder in which an individual has a significant delay in sleep onset time as compared to usual times in society, as well as difficulty in falling asleep at conventional times, but with the physiological structure of sleep and its efficiency preserved^1^^-^^3^. Its prevalence is estimated between 0.13% to 10% of the population, and it is more prevalent in young people, especially adolescents who experience sudden changes in their tasks, such as school and professional activities^4^. Furthermore, the onset of puberty and adolescence is characterized by a biological circadian delay in their sleep patterns^1^.

The main symptomatology derived from this cycle is excessive daytime sleepiness, which makes it difficult to wake up in the morning and to maintain concentration and motor activity throughout the day, reflecting directly on the work capacity and attention, with decreased cognitive and motor skills of patients. Thus, DSWPD has a considerable social and professional impact on these individuals^1^^,^^2^^,^^4^. Current treatments aim at changing the sleep phase to earlier times to increase sleep time and improve associated daytime deficiencies^1^^,^^2^.

Intense light emission or light therapy has been studied as an option to promote circadian alignment and sleep health. On patients, suffering from early awakening insomnia, light treatment during the early part of the sleep period seems to be capable to delay dim light melatonin onset and delay sleep start times, promoting circadian alignment and sleep health^5^. For DSWPD, light emission is considered as a therapeutic option, and it is represented by the high-intensity light emission in the patient’s eyes, in the morning when they wake up, through portable devices that can inhibit sleepiness. The response magnitude depends on the time, intensity, duration, and wavelength of light administration^1^^,^^2^^,^^4^. The pathophysiological mechanism of this therapy comprises inhibiting melatonin production through the pineal gland. In this way, an attempt is made to advance sleep onset times to more conventional and desired times, and to advance melatonin secretion times^2^^,^^3^^,^^6^.

Since there is no consensus in the literature on its efficacy and how it should be processed, this study aims to evaluate the effectiveness of light therapy for the treatment of delayed sleep-wake phase disorder.

## MATERIAL AND METHODS

### Study design

Systematic review.

### Search strategy

The search strategy was conducted using the electronic databases MEDLINE/PubMed, Virtual Health Library (VHL), PsycINFO, Web of Science and Scopus along with a hand search until September 2020. Through a combination of descriptors, including terms from Medical Subject Headings (MeSH), Health Science Descriptors (DeCS) and contractions of descriptors, it was obtained the fallowing search details: (bright[All Fields] AND (“phototherapy”[MeSH Terms] OR “phototherapy”[All Fields] OR (“light”[All Fields] AND “therapy”[All Fields]) OR “light therapy”[All Fields])) AND ((“sleep disorders, circadian rhythm”[MeSH Terms] OR (“sleep”[All Fields] AND “disorders”[All Fields] AND “circadian”[All Fields] AND “rhythm”[All Fields]) OR “circadian rhythm sleep disorders”[All Fields] OR (“delayed”[All Fields] AND “sleep”[All Fields] AND “phase”[All Fields] AND “syndrome”[All Fields]) OR “delayed sleep phase syndrome”[All Fields]) OR (“sleep initiation and maintenance disorders”[MeSH Terms] OR (“sleep”[All Fields] AND “initiation”[All Fields] AND “maintenance”[All Fields] AND “disorders”[All Fields]) OR “sleep initiation and maintenance disorders”[All Fields] OR “insomnia”[All Fields])).

References of the identified and selected articles by the search strategy were also investigated manually to add to the study and literature review. We contacted the corresponding authors of two studies not yet published but we did not get a response. It was used the PRISMA^7^ protocol as a guide for the systematic review.

### Inclusion and exclusion criteria

The inclusion criteria were: studies whose participants were diagnosed with insomnia or delayed sleep-wake phase disorder and treated only with morning light therapy, indicating the light intensity (lux) used, and investigations with a control group; studies published until 09/20/2020; studies in English or Portuguese; studies with participants aged 18 years or older; studies conducted only with human beings.

The exclusion criteria were: studies whose participants presented other comorbidities that justified the presence of insomnia or delayed sleep-wake phase disorder as neurological and/or psychiatric conditions; studies with pregnant women; studies without socioeconomic information; and studies that evaluated other disorders beyond the sleep spectrum.

### Identification and selection of studies

Two authors independently evaluated the identified articles by the initial search strategy according to inclusion and exclusion criteria. In case of divergence regarding studies’ inclusion or exclusion, the advisor had the deciding vote. The manual search followed the same selection principle.

### Extraction of data

The variables of interest extracted from selected articles through the eligibility criteria were the information concerning the authors, titles, the population sociodemographic profile (age, gender, and sex), the population of the light therapy and control groups, amount of lux applied, time of application, the total duration of therapy, and objective and subjective effects on sleep of the patients. We analyzed the quality of each study based on the Cochrane tool^8^. The items comprising the tool aim to assess the risk of bias in each study. They are: randomization (sequence generation), blind assessor, incomplete outcome data, selective outcomes, and intention to treat. Two authors classified each item as to the risk of bias as low risk, high risk, or unclear. In case of disagreement, a third author was consulted.

### Ethical aspects

This review registered in Prospero with number: 212016.

## RESULTS

**Figure 1:**
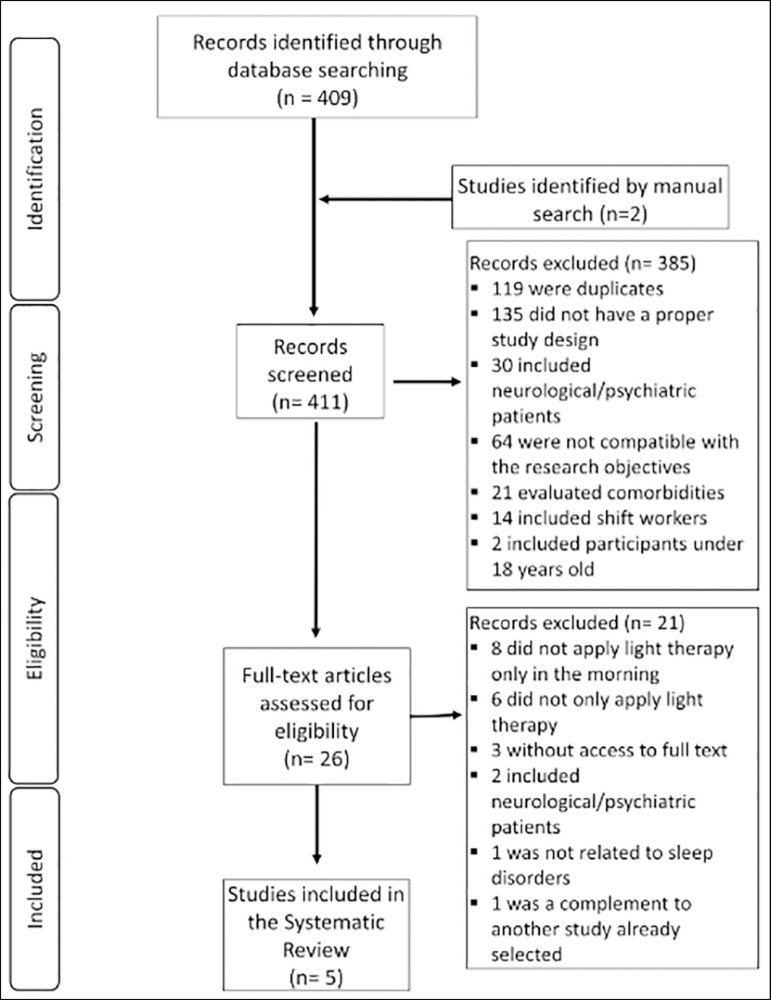
Flowchart of the study selection process.

**Figure 2:**
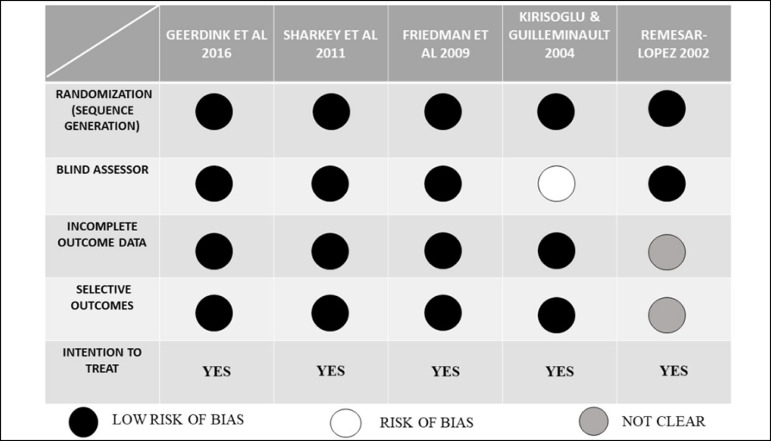
Assessment of the risk of publication bias - Cochrane tool.

During the studies selection, two authors evaluated titles and abstracts according to inclusion and exclusion criteria. From the 411 references collected, 26 were selected for a full reading. Of these, the following were excluded: one study because the patients did not present sleep disorders; one was an addendum to another study already included; two included patients with neurological and/or psychiatric disorders; eight applied light therapy out of the morning period; six did not contain samples or results of isolated light therapy; and three because of the impossibility of obtaining the entire studies, even after trying to contact the authors. Hence, for this systematic review, five articles were selected (Figure 1). The quality of each study was evaluated according to the Cochrane tool (Figure 2).

### Characteristics of participants

The main characteristics of the included studies are reported in Table 1. The samples ranged from 20^2^ to 39^9^ participants (n=140), considering only samples related to morning light therapy, including the control group. The mean age ranged from 21.4±6.5^9^ to 64.8±7^10^ years. All studies involved men and women, with a predominance of the female population. The studies did not report if there were comorbidities beyond those that could directly affect the participants’ sleep. Therefore, comorbidities were also excluded in our investigation to include the studies. The intervention duration ranged from 15^11^ to 84 days^12^.

**Table 1:** Methodological and characteristics of the studies included in the systematic review

Reference	Country/ Year	N/N*	Mean Age/Gender	Objective	Randomization	Intervention	Study Duration (in days)
Friedman et al ^12^	USA/2009	51/26	63,6 ± 7,1 M/F	Demonstrate the efficacy of light therapy in the insomnia treatment in older adults	The individuals were randomized into four treatment groups: dim light in the morning, dim light at night, bright light in the morning, and bright light at night.	Patients received approximately 4,000 lux, 15 minutes after waking up, for 45 minutes, associated with sleep hygiene	84/84**
Kirisoglu & Guilleminault ^10^	USA/2004	30/30	64,8 ± 7 M/F	Evaluate the difference in the efficacy of light therapy in the insomnia treatment	Random distribution of daily exposure groups, blind analysis of comparison data	Patients received, 10,000 lux for 20 or 45 minutes, 5 minutes after waking up, associated with sleep hygiene	60/60**
Remesar-Lopez ^2^	Brazil/2002	20/20	25,2 ± 11,3 M/F	Evaluate the effects of isolated and combined use of melatonin and light therapy	The volunteers were randomly divided into three groups: morning light therapy and placebo at night; placebo of morning light therapy and melatonin at night; and morning light therapy and melatonin at night.	The patients received 10,000 lux for 30 minutes, starting after verification of the minimum rectal temperature of each volunteer, separated into groups with and without melatonin	28/28**
Sharkey et al. ^11^	USA/2011	25/25	21,8 ± 3 M/F	Observe the effects of advanced sleep schedule	Participants were randomly assigned to groups to receive "blue" or "dim" light for 1h after waking each day.	Patients received 225 lux for 1 hour, just after waking up, with pre-established times to go to bed	15/6**
Geerdink et al ^9^	Netherlans 2016	39/39	21,4 ± 6,5 M/F	Analyze the efficacy of blue light pulses of compared to amber light pulses	Randomized, double-blind, placebo-controlled trial comparing pulses of blue light versus amber placebo light	Patients received blue light pulses (± 2,600 lux) and amber light pulses (320 lux) 30 minutes after waking up, with pre-established times to go to bed	30/9**

* Considering just sample that received light therapy in the morning and control

** Considering only the application days of light therapy

### Characteristics of selected studies

The study by Friedman et al. (2009)^12^ aimed to demonstrate the efficacy of light therapy in the insomnia treatment in older adults (63.6±7.1 years), improving the quality and time of sleep onset. The participants were recruited through a rigid evaluation to determine sleep disorders. They underwent polysomnography (PSG), obstructive sleep apnea screening, and assessment with sleep logs and a sleep physician. Thus, patients with possible insomnia secondary to other pathologies such as depression and dementia were excluded. After recruitment, the individuals were randomized into four treatment groups: dim light in the morning, dim light at night, bright light (light therapy) in the morning, and bright light at night. Both treatments in the morning consisted of 12 weeks, once a day, 15 minutes after waking up with 45 minutes of exposure. The light was calibrated to emit approximately 4,000 lux for the light therapy and 65 lux for the control group (dim light). All patients received sleep hygiene education.

Kirisoglu and Guilleminault (2004)^10^ assessed the difference in the efficacy of light therapy in the insomnia treatment in two methodologies: 20 minutes versus 45 minutes of application; in both cases, carried out 5 minutes after waking up, with 10,000 lux light therapy. Their inclusion and exclusion criteria were similar to the investigation of Friedman et al. (2009)^12^ since they selected patients over 60 years of age, both genders, evaluation by polysomnography and clinical examination, excluding those with any chronic disease or use of medications.

Remesar-Lopez (2002)^2^ proposed to assess the effects of isolated and combined use of melatonin and light therapy at the sleep onset time, on the variation of the time of dim light melatonin onset (DLMO), and on the phase of minimum core body temperature in volunteers with the diagnosis of delayed sleep-wake phase disorder. Twenty volunteers were selected and divided into three groups: morning light therapy and placebo at night (*photo group*); placebo of morning light therapy and melatonin at night (*mel group*); and morning light therapy and melatonin at night (*photo+mel group*), for four weeks. The patients were submitted to a clinical interview, as well as the use of actigraph, polysomnography, collect of plasmatic melatonin, and rectal temperature during the pre-treatment phase (observational) and the treatment phase, with two weeks of pre-treatment, taken as a comparison for the treatment phase. The isolated light therapy group received 10,000 lux for 30 minutes (after obtaining the minimum rectal temperature in the pre-treatment, informed by the investigator) and melatonin placebo at night. If for any reason, they were unable to use the device, sunlight was allowed for 30 minutes, extending to a maximum of 4 hours.

The control groups received treatment as follows: one group received morning light therapy combined with 3mg of melatonin, 5 hours before the beginning of the usual sleep time (*photo+mel group*); and the other group received a placebo of morning light therapy, with low-light exposure (900 lux) for 30 minutes and 3mg of melatonin, in the same way as the previous group (*mel group*). All groups, including the treatment group, followed some sleep hygiene recommendations, such as avoiding coffee and intense physical activity at night, besides keeping regular bedtimes^2^.

Sharkey et al. (2011)^11^ observed the effects of advanced sleep schedule, i.e., patients with subclinical criteria for delayed sleep-wake phase disorder had to go to bed earlier (on average 1 to 2.5 hours as compared the pre-treatment week) and had to be prepared to sleep out of their usual schedules, with prescribed sleep-wake schedules, 7.5 hours in bed, associated or not with the morning light therapy. The treatment group received a blue light emission of 225 lux (n=12) immediately after waking up for one hour. The control group used a low light of 1 lux (n=13). The intervention lasted six days.

Patients were instructed to maintain their usual sleep-wake schedules during the first week of monitoring (pre-treatment). There were no fixed bedtimes and wake times, as well as no instructions about exposure to light or dark. During the two weeks of pre-treatment and treatment, they were instructed to avoid naps and always sleep in their beds^11^.

During the treatment week, patients went to bed at fixed and advanced times (earlier) in the dark condition, 7.5 hours per night, avoiding light exposure. These times were defined individually, according to the nocturnal melatonin phase observed during the pre-treatment week and the commitments of each patient, and they were confirmed through actigraphy, sleep logs, and telephone calls. Thus, sleep-wake times were between 1 to 2.5 hours earlier than the average time observed during the week of pre-treatment, which also allowed the use of light therapy daily without interfering in social commitments^11^.

Geerdink et al. (2016)^9^ analyzed the efficacy of blue light pulses of 2,600 lux (n=18) compared to amber light pulses of 320 lux (n=21), in a home-setting protocol, both 30 minutes after waking up, associated with fixed and advanced sleep times, with pre-established schedules, similar to the study by Sharkey et al. (2011)^11^. The protocol was performed in 30 days, being 14 days of pre-treatment, only observational, without sleep restrictions and schedules; 9 days of treatment; and 7 days of post-treatment without sleep restrictions and light therapy. The predetermined schema of advancing time comprised a reduction of one hour every three days, during the treatment week, based on the times observed in the baseline week between days 4 and 10.

### Analysis of variables

#### Access to outcome

**Figure 3:**
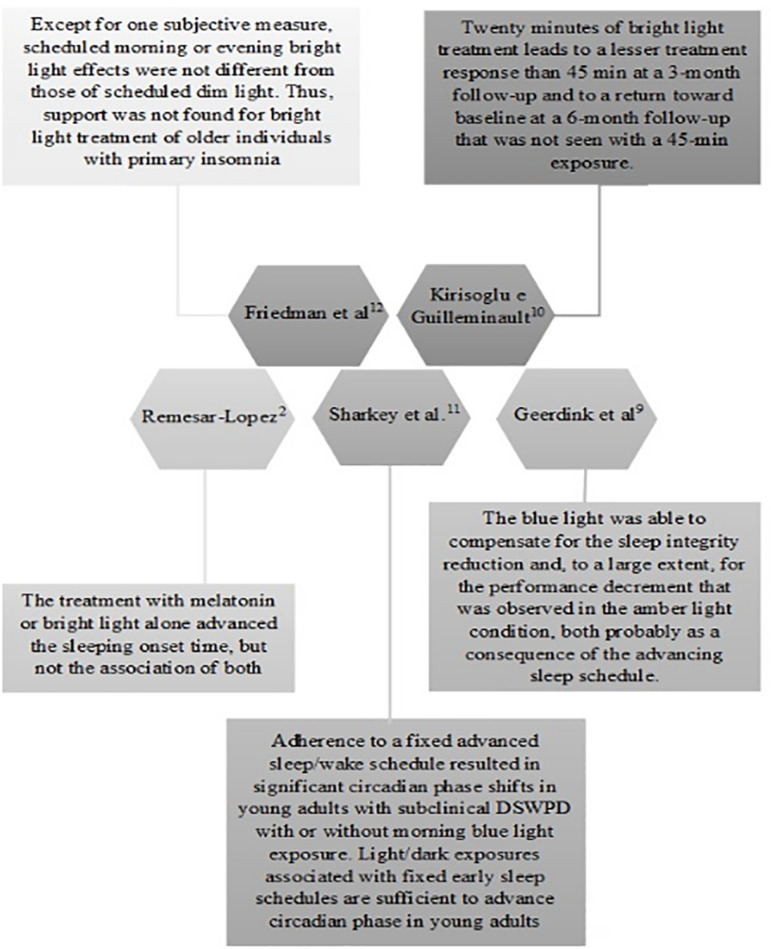
Conclusions of the analyzed studies.

The studies included in this review used objective and subjective measurements to evaluate treatment efficacy. All of them used sleep logs as subjective sleep evaluation. The main conclusions of the studies are described in Figure 3.

Friedman et al. (2009)^12^ used, as additional subjective evaluation for other parameters, questionnaires such as the Epworth sleepiness scale, the Spielman insomnia symptom questionnaire, the sleep hygiene scale, the sleep satisfaction scale, and the SF-36 quality of life questionnaire (physical and mental). Kirisoglu and Guilleminault (2004)^10^ used the visual analogue scale (VAS) to assess fatigue, the sleep disorders questionnaire, and the Epworth sleepiness scale. Remesar-Lopez (2002)^2^ used the profile of mood states (POMS), the state-trait anxiety inventory (STAI), and the morningness-eveningness questionnaire (MEC). In this latter study, the sleep log, used as a complementary tool to the actigraph for registering sleep, did not bring results of the variables apart from this monitor, like other studies.

The variables analyzed by Friedman et al. (2009)^12^ through the sleep logs were: total sleep time (TST), wake after sleep onset (WASO), sleep efficiency (SE), and time in bed (TIB). While Kirisoglu and Guilleminault (2004)^10^ studied sleep latency (SL) and total sleep time (TST), also through sleep logs. Although complementary to the actigraph, the sleep log analysis of Remesar-Lopez (2002)^2^ brought parameters such as “quality of sleep” and “how you felt when you woke up”, both obtained by marking on a 10cm ruler, drawn on the log.

Sharkey et al. (2011)^11^ used, as subjective evaluation, the sleep log, the morningness-eveningness questionnaire (MEQ), the state-trait anxiety inventory (STAI), the perceived stress scale (PSS), center for epidemiologic studies depression questionnaire (CES-D), positive and negative affect scale (PANAS), and 14 out of 25 patients completed a questionnaire on the experience felt during programming with fixed sleep schedules. However, similarly to Remesar-Lopez (2002)^2^, the sleep log data were compiled with actigraphy data.

Geerdink et al. (2016)^9^ used the sleep log, including sleepiness measurements in 5 and 30 minutes after waking up, using the Karolinska sleepiness scale (KSS). They also measured cognitive performance, which was analyzed using a minicomputer for a few days and at varying times throughout the study. However, the variables collected by the sleep logs did not figure in the study, nor was it made explicit whether the variables were compiled with actigraphy. Thus, for subjective sleep analysis, only the investigations by Friedman et al. (2009)^12^ and Kirisoglu and Guilleminault (2004)^10^ were included in Table 2.

**Table 2:** Subjective and Objective Pre and Post-treatment Evaluation

Reference	Type of evaluation		Total Sleep Time (min)		Sleep Latency (min)		Wake After Sleep Onset (min)		Sleep Efficiency (%)		Time in Bed (min)	
			Intervention	Control	Intervention	Control	Intervention	Control	Intervention	Control	Intervention	Control
	Subjective (Sleep log)	Pre	339,2±51,8	320,6±34	NR	NR	74±37,3	69,5±46,2	66,8±9,1	69,4±12,4	512,4±57,1	470,2±60,2
		Post	367,5±72,5	291,1±118,6	NR	NR	49,9±46	63,3±41,4	77±12,9	73,1±7,7	477,9±41,1	463,6±48,1
Frieldman^12^	Objective (Polysomnography)	Pre	336.3± 44.5	320.7± 62.8	NR	NR	88.8± 48.7	82.9±40.4	72.5±10.1	72.2±11.4	470.4± 42.3	443.8±48.5
		Post	345.5± 49.1	320.0± 69.8	NR	NR	86.4± 44.5	68.2±28.0	74.3± 8.4	74.1± 8.8	466.2± 53.0	428.9±66.2
	Objective (Actigraphy)	Pre	NR	NR	NR	NR	66,2±37,7	61,3±27,3	80,6±7	78,9±6,7	508,8±55,5	470,2±60,2
		Post	NR	NR	NR	NR	55,7±35,1	63±19,2	82,4±8,2	76,7±7,3	476,1±43,1	463,6±48,1
Kirisoglu & Guilleminault ^10^	Subjective (Sleep log)	Pre	322,4±15,6	328,5±11,8	47,1±6,4	45,5±7,1	NR	NR	NR	NR	NR	NR
		Post	411,2±12,6	342,8±23	16,6±5,6	38,1±7,4	NR	NR	NR	NR	NR	NR
		Pre	NR	NR	NR	NR	53±16	51,6±36,4	86,5±6,8	85,5±8,28	NR	NR
Geerdink et al. ^9^	Objective (Actigraphy)	Post	NR	NR	NR	NR	54,6±26,3	61,3±44,6	85,9±5,6	85±8,7	NR	NR

*NR = Not reported

We observed that all studies utilized actigraphy as an objective evaluation. The study by Friedman et al. (2009)^12^ was more detailed and also used polysomnography (Table 2), and plasma melatonin measurement in the laboratory, with serial measurements performed from 5 p.m. to 9 a.m., with <10 lux of ambient light). They assessed the*melatonin midpoint*(mean melatonin time - calculated as the mean of the crossing between the maximum and minimum hours, in quantity, within the 16 hours of collection);*duration of melatonin*(duration in hours of melatonin, defined as the time between the crossing of the maximum and minimum melatonin); and*circadian angle phase*(calculated by subtracting the midpoint of sleep, i.e., the mean of bedtimes values DLMO hours), before and after the intervention. Thus, the*circadian phase angle*predicts the magnitude of how much light can induce changes in the circadian phase. A*positive circadian phase angle*indicates that the sleep midpoint occurred after the melatonin midpoint^12^. The variables obtained through actigraphy were total sleep time (TST), wake after sleep onset (WASO), sleep efficiency (SE), and time in bed (TIB).

Remesar-Lopez (2002)^2^ and Kirisoglu and Guilleminault (2004)^10^ also used polysomnography, but only in the pre-treatment and in the screening phase of patients, respectively, and they did not provide data for comparison with post-treatment. Kirisoglu and Guilleminault (2004)^10^ evaluated total sleep time (TST) and sleep latency (SL); however, it was the only study that did not measure melatonin. Remesar-Lopez (2002)^2^ also evaluated the plasma melatonin measurement, reporting the DLMO and core body temperature (rectal), which showed the rhythmic parameters nadir: MESOR and amplitude. The variables analyzed by Remesar-Lopez (2002)^2^ were sleep onset time (SOT) and total sleep time (TST).

Sharkey et al. (2011)^11^ also used salivary melatonin measurement, determining the DLMO and*circadian phase angle*between DLMO and sleep time and the Daysimeter. This light-sensing device captured the level of radiation used at eye level and quantified light exposure while awake. Patients were also instructed to keep the Daysimeter on in the room while sleeping so that it was possible to quantify the level of ambient light. Its use was for observational and evidential purposes, in addition to ambient light measurement. The variables analyzed by Sharkey et al. (2011)^11^ were the total sleep time (TST) and the time when these patients went to bed (bedtime) and when they woke up (wake time), before and after treatment.

Geerdink et al. (2016)^9^ assessed salivary melatonin, evaluating the DLMO before, immediately after, and 7 days after the light therapy, and, like Sharkey et al. (2011)^11^, also used Daysimeter for the same purposes as the author mentioned above. The variables analyzed by Geerdink et al. (2016)^9^ were sleep latency (SL), total sleep time (TST), wake time after sleep start (WASO), number of interruptions, and sleep efficiency (SE). As did Sharkey et al. (2011)^11^ and Geerdink et al. (2016)^9^ also investigated bedtimes and wake times.

In addition to the difference in duration among interventions, Friedman et al. (2009)^12^ evaluated data immediately before and after the treatment, and Kirisoglu and Guilleminault (2004)^10^ evaluated before the therapy, 3 and 6 months after the intervention. The data shown in the study tables of Friedman et al. (2009)^12^ refer to the group that received morning light therapy (n=19), comparing pre- and post-intervention with the group that did not receive morning light therapy (low light, n=7). Since the purpose was to evaluate the change in sleep behavior of these patients in the long-term, the data used in the comparison tables, for the work of Kirisoglu and Guilleminault (2004)^10^, referred to before the intervention and 6 months after treatment, for the*45 minutes*group of light therapy as an intervention group (n=15) and the*20 minutes*group as a control group (n=15).

The investigation results of Remesar-Lopez (2002)^2^, compare the TTS variable of the treatment group with the two control groups; however, for comparison with the other selected studies of this review, the control group that we included in the tables was the one that received a placebo of light therapy and melatonin at night (*mel group*). Furthermore, the data presented refer to the period before the intervention (pre-treatment) and the end of the last week of intervention (fourth week of treatment).

The results reported by Geerdink et al. (2016)^9^ were demonstrated by comparing: the pre-treatment week (14 days of observation); immediately after the intervention (23^rd^ day, considering nine days of intervention); and post-intervention (30^th^ day, seven days after the intervention) to assess the long-term effect.

The work of Friedman et al. (2009)^12^ has the following limitations: the absence of a control group, which makes it impossible to determine whether the observed improvements were due to a “participation” effect or to sleep hygiene instructions. Besides, participants were recruited based on primary insomnia complaints and not on delayed sleep phase disorder, so the observed effects do not necessarily extend to DSWPD. Finally, the study was unable to show consistent treatment effects on objective sleep parameters, such as actigraphy and polysomnography. As Geerdink et al. (2016)^9^, performed the intervention in the domestic environment, their results were influenced by the inherent environmental variants. In this study, participants also had no long-term follow-up, making it impossible to determine therapeutic efficacy in this context. Kirisoglu and Guilleminault (2004)^10^, on the other hand, despite having analyzed the long-term results, limited their selection to only participants with insomnia, which means that their results do not necessarily have practical application for DSWPD.

In the study by Sharkey et al. (2011)^11^, the studied participants had no diagnosis of DSWPD, only subclinical characteristics, and they were not followed over time to determine the permanence of the results. Another limitation of this work is that it was conducted in a field setting and the participants were not observed directly during the intervention. Remesar-Lopez (2002)^2^, in turn, analyzed the effects of the therapy implemented over a year; however, the limitation was the absence of a control group and the small sample (n=20).

## DISCUSSION

In this systematic review using a sample of 140 participants, we found a heterogeneous effect of interventions carried out in different populations on sleep phase delay. Although the application of light therapy showed an advanced sleep time in all studies, this evaluation was not significant when comparing the intervention and control groups, as well as when comparing the pre-treatment periods (observational and taken as comparison parameters) and the treatment or post-treatment periods for those studies that used them. Only one author who did long-term follow-up reported stability of effects on the variables studied at three and six months post-treatment; however, sleep onset time was not considered by this author. Another study showed that only one patient reported permanence of effects at 12 months of follow-up. These data are in agreement with several similar previous studies^13^^-^^18^.

In most studies, despite an advance in sleep and wake times, these conditions were not accompanied by the statistically significant advance of the circadian phase, measured through the time of dim light melatonin onset (DLMO), either in the intervention group concerning the pre-treatment period or when compared to the control group. Only one study demonstrated a significant phase advance of DLMO, and even so, this effect did not last over time, returning to the baseline values of DLMO immediately after the treatment cessation. This aspect diverged from what was found by Fargason et al. (2017)^19^, who demonstrated an advance of DLMO in detriment of the variables collected by the actigraph in patients with ADHD, when they were submitted to two weeks of treatment with 10,000 lux, during 30 minutes, 3 hours after awakening.

Crowley and Eastman (2015)^16^ and Dewan et al. (2011)^20^ demonstrated that the greater the exposure time to phototherapy, the greater magnitude the changes in the secretion of melatonin have, being more efficient than to increase the luminous intensity. That reinforces the result from Kirisoglu and Guilleminault (2004)^10^ when they found better values for the group from 45 minutes when compared to the group from 20 minutes of exposure to 10,000 lux.

The symptomatology of DSWPD, i.e., delayed sleep timing beyond the social conveniences, was temporarily improved, but there were no changes to match in the laboratory. Moreover, in most studies, the benefits were not sustained over time.

Another relevant aspect observed by the authors was total sleep time (TST). Three of the five studies did not show significant changes, while the others presented different results (one raises the TST, and the other gives a decrease of the TST). This fact may have occurred because of an advance of the sleep onset time was accompanied by an advance of the wake-up time; hence it did not cause great differences in the TST.

An analysis of the subgroups according to the similarity of the methodologies used permits to state those studies that used a high amount of lux obtained effects that lasted during their respective post-treatment periods (both used 10,000 lux). The permanence of the effects for some patients can be explained as follows: after reaching the desired sleep time, these patients can reduce their exposure to light at night^21^, as well as increase their exposure to daylight, whether artificial or sunlight, since Dagan et al. (1991)^22^ apud Remesar-Lopez (2002)^2^ point out that exposure to sunlight can also affect the circadian phase.

As mentioned previously, in all studies, the variables of sleep onset/sleep time had steady progress, but without statistical significance when compared to the control group. Three of the four authors who considered the sleep time in their variables suggested that light therapy might not have been the main responsible for the time advance, but rather sleep hygiene and the programming of advanced and fixed bedtime, as was used in two of these studies. Light therapy was only a driving force of the results since the advances were greater in the intervention groups, but not differently significant between the groups. These investigations find similarities to others that used advanced sleep protocol with rigorous bedtime^13^^,^^16^^,^^18^^,^^23^^,^^24^Adelaide, South Australia.; Patients: 49 adolescents (mean age 14.6 ± 1.0 y, 53% males.

In general, light therapy has proved to be effective in the treatment of symptoms of DSWPD in the short-term. The management protocols should be clarified since the different works used different lux intensities, as well as varied duration and time of use. More concrete results were demonstrated in studies such as Rosenthal et al. (1990)^14^ and Lack et al. (2007)^15^, when they used 2,500 lux for 2 hours and 1 hour, respectively. However, similarly to the studies used in this review, the benefits were not sustained over time.

In summary, the main findings of the analyzed studies were: an advance in sleep onset time using light therapy in the morning and at night, but the same effect using light therapy in the morning and melatonin at night was not observed^2^; the blue light in the morning was not associated with more significant phase shifts than dim light exposure^11^; a substantially greater improvement in the 45-minute exposure condition versus 20 minutes^10^, subsequently, the variables returned to baseline in the 20-minute condition, but not in the 45-minute condition; there were changes in the subjective and objective evaluation of patients^12^; and the phase advance of melatonin rhythm was markedly greater in the blue light exposure group^10^.

Some limitations should be considered. The first one involves the samples size, which varies from 20 to 39 participants and the discrepancies in age (which ranged from 21.4 to 64.8 years). Thus, it is valid to consider the necessity of more extensive studies of this magnitude since all investigations included small samples.

Besides, the small number of studies that fulfilled the eligibility criteria limits the populations studied, which may reduce the external validity of the review as a whole. Another aspect is that the light therapy application methodology differed widely among the researches in terms of lux intensity, treatment duration, application time, and concomitant use of alternative treatments.

However, as not all studies used the same diagnostic criteria, there may be discrepancies in diagnosis. Nevertheless, the criteria used, whether DSM-V or ICDS, are applied in clinical practice, which suggests that they are well categorized. Thus, diagnoses, according to official criteria, would contribute to the validity of the results of future researches. The control groups were different, received different treatments, and not all of them were placebo-controlled.

Finally, the methodology for evaluating the results differed among the authors, with convergence concerning only the objective evaluation by actigraphy and the one variable (total sleep time). Besides, not all studies fully exposed what they found in some subjective measurements, such as the sleep log.

The strong points of this review are the inclusion of only randomized clinical trials, the absence of search restrictions for publications only in English, and the evaluation of each light therapy intervention independent of its results. It is worth mentioning that this is the first systematic review focused on light therapy for the treatment of delayed sleep-wake phase disorder.

## CONCLUSION

This review demonstrates that light therapy, in general, could be effective in reducing sleep onset and wake times, as well as improving sleep latency and quality. Given the higher number of complaints of sleep problems in today’s society, efforts are required to reduce the systemic harmful effects of sleep disorders. In any case, it should be considered that there is still a lack of conclusive data for the treatment of DSWPD, reinforcing the importance of further studies of this nature.

## References

[1] Micic G, Lovato N, Gradisar M, Ferguson SA, Burgess J, Lack L (2015). The etiology of delayed sleep phase disorder. Sleep Med Rev.

[2] Remesar-Lopez AJ (2002). Sleep delayed phase syndrome: the effects of melatonin and bright light
alone, or in combination.

[3] Shirayama M, Shirayama Y, Iida H, Kato M, Kajimura N, Watanbe T (2003). The psychological aspects of patients with delayed sleep phase
syndrome (DSPS). Sleep Med.

[4] Magee M, Marbas EM, Wright KP, Rajaratnam SMW, Broussard JL (2016). Diagnosis, cause, and treatment approaches for delayed sleep-wake
phase disorder. Sleep Med Clin.

[5] Figueiro MG (2015). Individually tailored light intervention through closed eyelids
to promote circadian alignment and sleep health. Sleep Health.

[6] Martinez D, Lenz MCS, Menna-Barreto L (2008). Diagnosis of circadian rhythm sleep disorders. J Bras Pneumol.

[7] Liberati A, Altman DG, Tetzlaff J, Mulrow C, Gotzsche PC, Ioannidis JP (2009). The PRISMA statement for reporting systematic reviews and
meta-analyses of studies that evaluate healthcare interventions: explanation
and elaboration. BMJ.

[8] Higgins JPT, Altman DG, Gotzsche PC, Jüni P, Moher D, Oxman AD (2011). The Cochrane Collaboration's tool for assessing risk of bias in
randomised trials. BMJ.

[9] Geerdink M, Walbeek TJ, Beersma DGM, Hommes V, Gordijn MCM (2016). Short blue light pulses (30 min) in the morning support a
sleep-advancing protocol in a home setting. J Biol Rhythms.

[10] Kirisoglu C, Guilleminault C (2004). Twenty minutes versus forty-five minutes morning bright light
treatment on sleep onset insomnia in elderly subjects. J Psycosom Res.

[11] Sharkey KM, Carskadon MA, Figueiro MG, Zhu Y, Rea MS (2011). Effects of an advanced sleep schedule and morning short
wavelength light exposure on circadian phase in young adults with late sleep
schedules. Sleep Med.

[12] Friedman L, Zeitzer JM, Kushida C, Zhdanova I, Noda A, Lee T (2009). Scheduled bright light for treatment of insomnia in older
adults. J Am Geriatr Soc.

[13] Gradisar M, Dohnt H, Gardner G, Paine S, Starkey K, Menne A (2011). A randomized controlled trial of cognitive-behavior therapy plus
bright light therapy for adolescent delayed sleep phase
disorder. Sleep.

[14] Rosenthal NE, Joseph-vanderpool JR, Levendosky AA, Johnston SH, Allen R, Kelly KA (1990). Phase-shifting effects of bright morning light as treatment for
delayed sleep phase syndrome. Sleep.

[15] Lack L, Wright H, Paynter D (2007). The treatment of sleep onset insomnia with bright morning
light. Sleep Biol Rhythm.

[16] Crowley SJ, Eastman CI (2015). Phase advancing human circadian rhythms with morning bright
light, afternoon melatonin, and gradually shifted sleep: can we reduce
morning bright-light duration?. Sleep Med.

[17] Watanabe T, Kajimura N, Kato M, Sekimoto M, Takahashi K (1999). Effects of phototherapy in patients with delayed sleep phase
syndrome. Psychiatry Clin Neurosci.

[18] Cole RJ, Smith JS, Alcalá YC, Elliott JA, Kripke DF (2002). Bright-light mask treatment of delayed sleep phase
syndrome. J Biol Rhythm.

[19] Fargason RE, Fobian AD, Hablitz LM, Jodi R, White BA, Cropsey KL (2017). Correcting delayed circadian phase with bright light therapy
predicts improvement in ADHD symptoms: a pilot study rachel. J Psychiatr Res.

[20] Dewan K, Benloucif S, Reid K, Wolfe LF, Zee PC (2011). Light-induced changes of the circadian clock of humans:
increasing duration is more effective than increasing light
intensity. Sleep.

[21] Jet VII, Boulos Z, Campbell SS, Lewy AJ, Terman M, Dijk D (1995). Light treatment for sleep disorders: consensus report. VII. Jet
lag. J Biol Rhythm.

[22] Dagan Y, Tzichinsky O, Lavie P (1991). Sunlight treatment for delayed sleep phase syndrome: a case
report. Sleep.

[23] Saxvig IW, Wilhelmsen-Langeland A, Pallesen S, Vedaa O, Nordhus IH, Bjorvatn BB (2014). A randomized controlled trial with bright light and melatonin for
delayed sleep phase disorder: effects on subjective and objective
sleep. Chronobiol Int.

[24] Danilenko KV, Cajochen C, Wirz-Justice A (2003). Is sleep per se a zeitgeber in humans. J Biol Rhythms.

